# The differential impact of friendship on cooperative and competitive coordination

**DOI:** 10.1007/s11238-020-09763-3

**Published:** 2020-07-06

**Authors:** Gabriele Chierchia, Fabio Tufano, Giorgio Coricelli

**Affiliations:** 1 Institute of Cognitive Neuroscience, University College London, London, UK; 2 Center for Mind/Brain Science, University of Trento, Trento, Italy; 3 School of Economics, University of Nottingham, Sir Clive Granger Building, University Park, Nottingham, NG7 2RD UK; 4 Department of Economics, University of Southern California, Los Angeles, USA

**Keywords:** Coordination, Entry game, Friendship, Strategic complementarity, Strategic substitutability, Stag-hunt game, Strategic uncertainty

## Abstract

Friendship is commonly assumed to reduce strategic uncertainty and enhance tacit coordination. However, this assumption has never been tested across two opposite poles of coordination involving either strategic complementarity or substitutability. We had participants interact with friends or strangers in two classic coordination games: the stag-hunt game, which exhibits strategic complementarity and may foster “cooperation”, and the entry game, which exhibits strategic substitutability and may foster “competition”. Both games capture a frequent trade-off between a potentially high paying but uncertain option and a low paying but safe alternative. We find that, relative to strangers, friends are more likely to choose options involving uncertainty in stag-hunt games, but the opposite is true in entry games. Furthermore, in stag-hunt games, friends “tremble” less between options, coordinate better and earn more, but these advantages are largely decreased or lost in entry games. We further investigate how these effects are modulated by risk attitudes, friendship qualities, and interpersonal similarities.

## Introduction

Coordination problems arise widely in social and economic contexts: from teams in the workplace (e.g., Lazear and Shaw 2007) to organizations (e.g., Milgrom and Roberts 1992); from collective actions (e.g., Chwe 2013) to macroeconomics (e.g., Cooper and John 1988).

From a game-theoretic perspective, coordination problems are characterised by multiplicity of Nash equilibria. As such, they pose a problem of equilibrium selection, which has been suggested to constitute “the most difficult problem in game theory” (Camerer 2003, p. 336). Most of the experimental research to date on equilibrium selection in coordination games has focused on how structural features (e.g., payoffs) of coordination games affect equilibrium selection and, consequently, the potential of social closeness as a coordination devise has remained largely unexplored (e.g., Camerer 2003; Devetag and Ortmann 2006).

Economics has traditionally assumed a parsimonious “social void” of homogeneous individuals (Charness et al. 2007a, b),^1^ whereas other social sciences tend to explain social behaviour by assuming that individuals are heterogeneous along various important dimensions such as their degree of relatedness (e.g., Hamilton 1964), their interpersonal similarities (e.g., McPherson et al. 2001), or their group membership (e.g., Tajfel and Turner 1979). These factors portray the notion of a social space in which, since very early infancy (e.g., Meltzoff 2007), across cultures (e.g., Apicella et al. 2012), and even across species (e.g., Massen and Koski 2014), tuning behaviour to the “social closeness” of others seems to be the rule rather than the exception in social interactions. Here, we turn to such a social space and ask whether social closeness, and friendship as a paradigmatic instantiation of closeness, may play a role as a coordination device.

So far, a number of experimental studies have shown that many different forms of social closeness impact significantly on economic decisions, generally by increasing prosocial behavior.^2^ Friendship makes no exception: several economic experiments demonstrate how friendship strongly predicts trustworthiness (Glaeser et al. 2000); has positive effects on microcredit repayments (Abbink et al. 2006); boosts reciprocal behaviour (Reuben and van Winden 2008); enhances dictator giving (Leider et al. 2009; Goeree et al. 2010); increases favoritism in variants of trust games (Brandts and Solà 2010). However, no study has asked whether the positive association between friendship and coordination is stable across opposite families of games, namely games of strategic complements and substitutes.

Games of strategic complements involve strategies that mutually enforce one another: that is, players have incentives to *match* their actions. For instance, putting in a few extra hours of work to complete your part of a group task on time may be worthwhile for everyone only if the other colleague does the same, and wasteful otherwise. By contrast, games of strategic substitutes involve strategies that offset one another, and consequently, players have incentives to *mismatch* their actions. For instance, co-workers might prefer to run their computations on the most powerful server, but if all do so at the same time, this could lead to a longer computational time for everyone. Thus, one should only use the most powerful server if he or she expects the other will *not* do so and vice versa.

Similar game structures extend to a variety of situations involving more than two players. Consider a case of strategic complements as the decision of joining a protest. The chances of a successful protest increase with the number of people joining it; however, joining is risky, because one would not want to protest alone. Consider now a case of strategic substitutes as firms’ decisions to bring their product to a target market: it could be profitable if not too many firms do the same, to avoid a price war. Notably, strategic complements are known to foster cooperation (e.g., Camerer and Fehr 2006; Potters and Suetens 2009), while strategic substitutes involve finite resources which are conducive to competition (e.g., Camerer and Lovallo 1999).

Hoffman et al. (1996) define social closeness as “the *degree of reciprocity* that subjects *believe* exist within a social interaction” (p. 654—italics added). Based on this, we conjectured that friends, relative to strangers, might be at an advantage when they are to coordinate on options that mutually benefit one another (i.e., matching choices in one-shot games with strategic complements), but that this advantage might be reduced—or even reversed—when they are to break the “degree of reciprocity” and coordinate on opposite choices (i.e., mismatching choices in one-shot games with strategic substitutes). For example, suppose that, out of love for a friend (e.g., player’s prosocial preference), a player wants to pick the option that most benefits their friend and, by expecting that their love will be *reciprocated*, believes their friend will do the same (e.g., player’s beliefs). Under strategic complements, this is clearly optimal given that players have incentives to match their actions, and both preferences and beliefs point in the same direction. On the other hand, under strategic substitutes, preferences and beliefs can run against one another: out of love for their friend, a player may want to leave the most profitable option to their friend, but if she/he believes their friend will do the same, then no one will take advantage of the most profitable option, which is clearly suboptimal. This dilemma is strongly reminiscent of the following considerations by Karl Popper: “That love as such may be unable to settle a conflict can be shown by considering a harmless test case, which may pass as representative of more serious ones. Tom likes the theatre and Dick likes dancing. Tom lovingly insists on going to a dance, while Dick wants for Tom’s sake to go to the theatre. This conflict cannot be settled by love; rather, the greater the love, the stronger will be the conflict.” (Popper 1945/2011, p. 441). In synthesis, under strategic complements, prosocial preferences and beliefs act synergistically, while this need not be the case for strategic substitutes.

To investigate the impact of social closeness in coordination games with strategic complements and substitutes, we have participants interact with either a friend or a stranger in two classic two-player games with real monetary payoffs: in “stag-hunt” games, due to the existence of strategic complements, players have the incentive to *match* their choices; conversely, in “entry” games, due to the existence of strategic substitutes, players have the incentive to *mismatch* their choices. In both stag-hunt and entry games, subjects face the same potential monetary payoffs. Specifically, they face a binary decision between an option involving *uncertainty* (i.e., yielding either $15.00 or $0, depending on what the other chooses), henceforth referred to as the “UP” action (short for ‘Uncertain Payoff’), and a lower paying but safe alternative (e.g., worth $7.50 for sure, regardless of what the other chooses), henceforth referred to as “SP” (short for ‘Sure Payoff’) action. In both games, the UP action thus requires coordinating with others, while the SP does not. The only difference between games is the consequence of choosing the UP action. In the stag-hunt game, players can jointly obtain the highest payoff (equal to $15) only if they both choose UP; in other words, the highest payoff requires a player to opt for UP and their opponent to *match* such a choice. By contrast, in the entry game, a player receives the highest payoff of $15 if they choose UP, while their opponent does not, namely the opponent needs to *mismatch*. To isolate the effect of social closeness from learning, here, we focus on one-shot games.

In such a framework, we define “strategic uncertainty” as uncertainty related to the players’ behaviour in a situation with interdependent decisions (Brandenburger 1996). Following Heinemann et al. (2009), we operationalise and measure strategic uncertainty as to the probability of choosing the UP action (such that a higher probability of choosing actions involving uncertainty reveals lower strategic uncertainty). To control for individual risk attitudes, we conduct a lottery task modelled as a game against ‘Nature’ with the same potential payoffs as those featured in the coordination games. As possible mediational factors of the impact of friendship on strategic uncertainty, we inspect friendship quality, perceived interpersonal similarities, and the frequency of past interactions, which have been shown to be an adequate proxy for future interactions (Zhang 2001). Finally, in addition to strategic uncertainty, we investigate how friendship affects prosocial choices; other coordination-related measures (such as the degree to which subjects “tremble” by switching back and forth between uncertain and safe actions); and the rates of expected coordination and payoffs.

We find that, relative to strangers, friends are more likely to choose options involving uncertainty in stag-hunt games, but the opposite is true in entry games. Moreover, friendship increases prosocial behaviour in both games, but the effect is much smaller for entry games. Friends coordinate better, “tremble” less and earn more than strangers in stag-hunt games, but these advantages are either decreased or are entirely lost in entry games. These findings are robust to controlling for participants’ risk attitudes, friendship quality, friends’ perceived similarities, and frequency of past interactions. Taken together, these results suggest that the impact of social closeness on coordination problems is clear and beneficial in games involving strategic complements, but that this is not the case in games with strategic substitutes.

The remainder of the paper is as follows. Section 2 introduces the study methods. Section 3 reports on the results. Section 4 discusses the implications of the empirical evidence. Section 5 concludes.

## Methods

### Tasks

#### Coordination games

The experiment has two parts, which participants encounter in sequence before taking the post-experimental questionnaire.^3^ In the first part, participants play two distinct two-player coordination games: stag-hunt games and entry games. The payoff matrixes for the stag-hunt games and for the entry games are respectively detailed in Table 1. The payoffs represent dollar amounts. Each participant plays 20 variants of each game and each variant is characterised by a different dollar value of X $\in \left\{0, 1, 2, 3, 4, 5, 6, 6.5, 7, 7.5, 8, 8.5, 9, 9.5, 10, 11, 12, 13, 14, 15\right\}$ (Nagel et al. 2017). Finally, each variant is played twice: once with a stranger and once with a friend (amounting to 80 decisions in total). Therefore, this experiment presents a within-subject design with two treatments: the *Stranger* treatment and the *Friend* treatment.

**Table 1:** Coordination games

	Column player				Column player		
		SP	UP			SP	UP
Row player	SP	X, X	X, 0	Row Player	SP	X, X	X, 15
	UP	0, X	15, 15		UP	15, X	0, 0
	a. Stag-hunt game				b. Entry game		

As we are interested in one-shot games, participants do not receive any feedback on game plays until the end of the experimental session. Moreover, to reduce excessive task switching, the stag-hunt games and the entry games are played in separate blocks, the order of which is counterbalanced across participants. Within each block, the level of social closeness (i.e., friend vs. stranger) and the values of the sure payoff X are randomized without replacement. Therefore, each block entails 40 decisions: 20 decisions (one per value of X) having as counterpart a friend and 20 decisions (again, one per value of X) having as counterpart a randomly selected stranger (with replacement).

For each game play, participants view the dollar value of the sure payoff (e.g., $8.50) on one side of their computer screen (labelled “A”), and the fixed $15 on the other (labelled “B”) (the sides are randomized). Thus, the two coordination games visually differ only by what is written next to the uncertain $15 option. Stag-hunt games have the following text: “$15.00 only if your counterpart chooses B, 0.00 if your counterpart chooses A”; entry games simply invert the positions of A and B in the text: that is, “$15.00 only if your counterpart chooses A, 0.00 if your counterpart chooses B”. Participants are informed about whom they are matched with for their current decision. Specifically, they either read “You are matched with a stranger” or “You are matched with [friend’s name]”, followed by this text: “You are both reading these same instructions. You both have to choose between the following two options: A or B. Which one do you prefer?” A screenshot for the stag-hunt game is reproduced in Appendix B (Fig. 2).

Participants are informed that for *each* game play in the stranger treatment, they will be matched with a randomly selected participant from the experimental session: this could be a different person each time or the same person, but participants are told that the stranger cannot be their friend. Participants are also informed that, at the end of the experimental session, one of their decisions will be randomly drawn, and that they will be paid according to the outcome of that decision. For example, if a decision from the stranger treatment is drawn, then participants are randomly paired with a non-friend counterpart and paid according to their actual choices.

#### Lottery task

After playing the coordination games, in the second part of the experiment, subjects take part in a lottery task to elicit individual risk attitudes, which have been shown to correlate with strategic uncertainty (Heinemann et al. 2009; Chierchia et al. 2018). The lottery task is set up as a game against ‘Nature’ resembling the above coordination games: participants choose between the sure payoff option SP and the risky-payoff option RP; if chosen, option SP yields the sure payoff X, regardless of the state of Nature. By contrast, option RP could lead to $0 if the state of Nature is A or to the highest payoff equal to $15 if the state of Nature is B. Participants know that the state of Nature will be determined by a blind lottery draw yielding A or B with a 50/50 probability. The payoff matrix of the lottery task is reported in Table 2. The sure payoff X assumes the exact same 20 values used for the coordination games. Therefore, participants are expected to make 20 decisions (i.e., one for each of the 20 dollar values assigned to SP) in the lottery task.^4^

**Table 2:** Lottery task: game against nature

		State of nature	
		A	B
		*p* = 1/2	(1 − *p*) = 1/2
Player	SP	X	X
	RP	0	15

A screenshot of the lottery task is reproduced in Appendix B (Fig. 3). To further stress the difference between the lottery task and the coordination games, at the start of each experimental session, we draw participants’ attention to an empty opaque box in which we openly place one yellow ball and one blue ball. It is then explained to participants that, at the end of the session, a single ball will be blindly drawn, and that the lottery-task payoff will depend on which one of the two balls is drawn. Visually, in each lottery choice, the colour of the winning ball is randomized (i.e., in some lottery choices participants are asked to bet on “blue” while in others to bet on “yellow”).

### Post-experimental questionnaires

Following the lottery task, participants complete a post-experimental questionnaire consisting of two main sets of items: those of the McGill Friendship Questionnaire (“MFQ” for short—Mendelson and Aboud 1999) and those of a novel “similarity measure”. Specifically, as a robustness check of our treatment manipulation, participants respond to the MFQ tapping into several dimensions of friendship quality.^5^ Moreover, since interpersonal similarities are one of the best-known predictors of friendship formation (Montoya et al. 2008) and have been shown to play a role in tacit coordination (Chierchia and Coricelli 2015), we measure participants’ perceived similarities with their friends. Adapted from Chierchia and Coricelli (2015), our similarity measure consists of 20 person-descriptive words (e.g., funny, disciplined, opinionated, etc.—see Appendix D for a complete list of words and screenshot of the task) that participants rate on a continuous scale ranging from − 50 to + 50. For each word, the rating is made twice: the first time, participants rate how well the word describes themselves (“self-ratings”); the second time, they rate how well it describes the friend that is sitting in the lab with them (“friend ratings”). In both cases, the value of “0” on the scale represents how well the word describes an “average” student from their own university. We use the Pearson’s correlation between self-rating and friend rating as a “perceived similarity measure”, given that it measures the degree to which friends think that they are more likely than the average student from their own University to have similar traits.^6^

Finally, as a possible proxy for future interactions, we ask participants how frequently they met their friends in the recent past (Zhang 2001). We do this by means of a single question: “During the past 6 months how regularly have you seen [friend’s name], on average?” Participants respond by selecting one of the following options: “every day”; “every four days”; “about once a week”; “about once every other week”; “about once a month”, which we code respectively from 5 to 1 to generate our variable *frequency of past interactions*.^7^

### Participants

Seventy-eight participants took part in the study across four experimental sessions conducted at the Los Angeles Behavioural Economics Laboratory (“Label”) of the University of Southern California. Students from a wide range of academic disciplines were recruited by both ORSEE (Greiner 2015) and flyers. The average duration of each session was 84 min. Participants were paid individually and anonymously at the end of each experimental session.^8^

Participants were required to bring a non-romantic friend to the experimental session. Upon arrival, they were randomly assigned to individually shielded computer cubicles. Instructions were read aloud and followed on individual handouts. Then, participants answered several control questions to ensure their understanding of the instructions and they could progress to the next stage only after providing the correct answers.

While participants were completing the post-experimental questionnaires, we downloaded their responses from the Qualtrics website and ran an in-house script to randomly match the participants; select at random the decision relevant for payment and determine participants’ experimental earnings according to their actual decisions. All procedures were approved by the local ethical committee.

### Measures and hypotheses

#### UP actions

Our main interest lies in understanding the effects of friendship on strategic uncertainty across coordination games of strategic complements and strategic substitutes. Thus, we first construct the variable “UP action” by coding choices as 1, if the UP action is chosen, and 0 otherwise; then, we operationalise strategic uncertainty as the probability of choosing UP actions (such that a higher probability of choosing UP actions reveals lower strategic uncertainty). We hypothesise that friendship may decrease strategic uncertainty in stag-hunt games (i.e., higher probability of UP actions), but not necessarily in entry games, where friendship may even increase strategic uncertainty (i.e., lower probability of UP actions).

#### Prosocial actions

A natural question is whether these effects of friendship on strategic uncertainty are entirely driven by prosocial preferences. As argued in the introduction, prosocial preferences for one’s friend (e.g., love) may induce friends to choose the UP action more frequently than strangers in the stag-hunt game and the SP actions in the entry game. We thus code both these actions (with a value of “1”) into the “prosocial action” dummy variable. We hypothesise that if friendship only operates through a prosocial preference channel (i.e., holding beliefs constant across games), it will equally increase the probability of prosocial actions in both games. Any differential impact of friendship on prosocial actions across games suggests that beliefs may at least partially mediate the effect of friendship on strategic uncertainty.

#### Trembling

“Trembling” refers to subjects switching back and forth from the UP to the SP actions (or vice versa) across game plays. Trembling is likely to evidence inconsistent decisions in stag-hunt games, while it is additionally compatible with increased reasoning in entry games (Chierchia et al. 2018; Nagel et al. 2017). We hypothesise that friendship would decrease trembling in the stag-hunt, but not in the entry game. Thus, to construct our trembling variable, we proceed as follows: for each game, we first order the 20 variants in ascending order, based on the sure payoff value; then, we dummy code each variant (with the exclusion of the first variant which by construction has $0 sure payoff) with a “1” if the choice had changed relative to the previous variant, and with a “0” if it had not.

#### Coordination and payoff rates

As performance-related measures, we focus on coordination and payoff rates. To compute coordination rates we proceed as follows. For the Friend treatment, we calculate for each participant in the stag-hunt game (resp. entry game) the percentage of times they matched (resp. mismatched) their choices with their friend across the twenty game variants. For instance, if participant “*i*” matched (resp. mismatched) their choices with their friend 18 out of 20 times, then *i*’s percentage is 90% and the coordination rate is thus 0.90. On the other hand, for the Stranger treatment, we compute for each participant in the stag-hunt game (resp. entry game) the percentages of times they could match (resp. mismatch) their choices with *any other* stranger playing the same game variant. We do so to reduce the noise inherent in the player random matching of the Stranger treatment. Then, we average across the percentages from each variant to calculate the coordination rate. For instance, if participant “*j*” in the Stranger treatment chooses the UP action in a stag-hunt game variant with a $5 sure payoff, and 78% of the other stranger players choose the UP action on that same variant, then *j*’s percentage of matching (resp. mismatching) actions for the $5 sure payoff variant is 78%. In the Entry game, everything else being equal, if *j* chooses the SP action instead of the UP action, their coordination rate is (1–0.78) = 0.22. We then average over the percentages so obtained across all the 20 game variants in the Stranger treatment to compute the coordination rate.

Notably, it can be easily shown that coordination rates and payoff rates may markedly differ (e.g., consider the case of full coordination on UP actions vs. full coordination on SP actions: these two cases would imply different payoffs). We thus proceed to also computing payoff rates as follows: if a participant chooses the UP action, the payoff is simply the maximum payoff (i.e., $15.00) multiplied by the coordination rate; while if a participant chooses the SP action, their payoff is unconditionally the sure payoff value (i.e., in the example above: $5). In this fashion, we computed payoff rates for each treatment and proceeded to investigate how they were affected by friendship. We hypothesise that friendship could increase coordination and payoff rates in the stag-hunt game, but less so in the entry game.

### Statistical and econometric analyses

Strategic uncertainty, prosocial behavior, as well as trembling are analysed using generalized mixed effect logistic regressions, clustering data at the participant level. As predictors, we use sure payoff, game and friendship, as well each possible two-way interactions between these factors (Tables 3,4 and Appendix C—Tables 5, 6, 7). In line with the previous literature on strategic uncertainty (e.g., Chierchia et al. 2018), we also inspect whether the hypothesised effects of friendship are robust to controlling for risk attitudes, as measured by the proportion of risky actions chosen in the lottery task. We additionally inspect the individual and aggregate role of the three potential mediators of the friendship effect on strategic uncertainty: perceived similarity, friendship quality, and frequency of past interactions (models 1–3, Table 4). Coordination and payoff rates are analysed with OLS regression. These latter regressions include the same predictors of previous models, but they omit the sure payoff variable as they focus on averages computed across variants with different payoff values (Appendix C, Table 7). (All of our econometric results go through when nesting participants within their friendship pairs.)

**Table 3:** Investigating UP actions: mixed-effects logistic regressions

Estimation method: mixed-effects logistic		
Controls for individual effects: clustering		
Dep. variable: UP action (dummy)		
	Model 1	Model 2
Sure payoff	− 0.251*** (0.015)	− 0.251*** (0.015)
Stag-hunt game (dummy)	2.418*** (0.187)	2.417*** (0.187)
Friend counterpart (dummy)	− 0.658*** (0.154)	− 0.658*** (0.154)
Stag-hunt game*Sure payoff	− 0.115*** (0.019)	− 0.115*** (0.019)
Friend counterpart*Sure payoff	0.048*** (0.018)	0.048*** (0.018)
Stag-hunt game*Friend counterpart	1.732*** (0.148)	1.732*** (0.148)
Risk		2.478*** (0.647)
Constant	1.469*** (0.160)	0.425 (0.311)
No. observations	6196	6196
No. individuals	78	78
Log-likelihood	− 2904.980	− 2898.199

(Robust) Standard errors are in parentheses

***Significance at the 1 percent level

**Table 4:** Friendship quality as mediational factor: mixed-effects logistic regressions

Estimation method: mixed-effects logistic Controls for individual effects: clustering Dependent variable: UP action (dummy)			
	Model 1	Model 2	Model 3
Sure payoff	− 0.209*** (0.016)	− 0.208*** (0.016)	− 0.208*** (0.016)
Stag-hunt game (dummy)	1.665*** (0.528)	1.722*** (0.533)	1.706*** (0.535)
Stag-hunt game*Sure payoff	− 0.136*** (0.031)	− 0.140*** (0.031)	− 0.142*** (0.031)
MFQ index	− 0.021 (0.098)	− 0.005 (0.094)	0.039 (0.116)
MFQ index*Stag-hunt game	0.459*** (0.076)	0.485*** (0.078)	0.590*** (0.100)
Similarity index		− 0.821** (0.386)	− 0.823** (0.383)
Similarity index*Stag-hunt game		− 0.567* (0.340)	− 0.560 (0.342)
Frequency of past interactions			− 0.071 (0.112)
Frequency of past interactions*Stag-hunt game			− 0.163* (0.095)
Constant	0.968 (0.635)	1.098* (0.612)	1.091* (0.607)
No. observations	3112	3112	3112
No. individuals	78	78	78
Log-likelihood	− 1325.301	− 1320.408	− 1318.275

(Robust) Standard errors are in parentheses

***Significance at the 1% level

**Significance at the 5% level

^*^Significance at the 10% level

## Results

### Friendship and strategic uncertainty

**Fig. 1:**
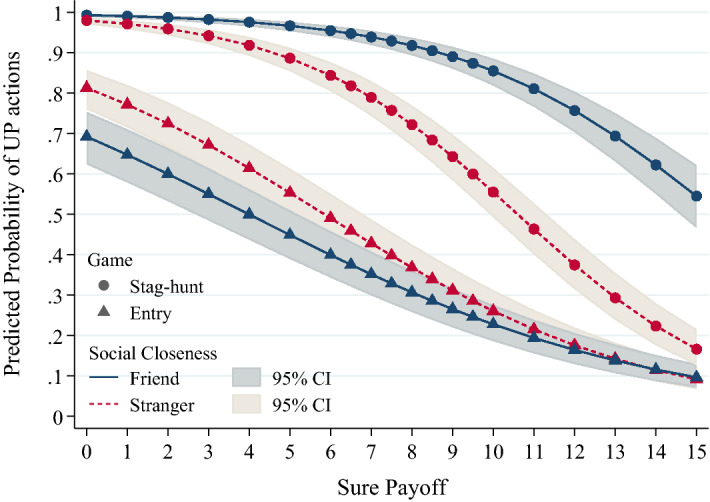
Impact of friendship on tacit coordination. Curves represent predicted probabilities of choosing UP actions (*y*-axis) across different values of a sure payoff (*x*-axis), when interacting with either friends (blue solid lines) or strangers (red dashed lines) in both stag-hunt games (circle marker) and entry games (triangle marker). The predicted probabilities were obtained from Model 1 mixed-effects logistic regression. Error bands represent 95% confidence bands of the fixed effects

**Fig. 2:**
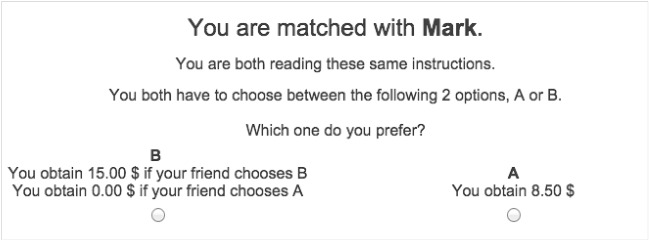
Screenshot of the coordination game. In this example, a participant is taking part in a “stag-hunt game, in the friendship condition” (Sect. 2). Before taking part in the games, participants were asked to write the name of their friend, which then reappeared in the friendship condition (in this example the participant’s friend’s name is “Mark”). In the stranger condition, one’s friend’s name was replaced with “a stranger”. In an “entry game”, in the “B” option, the letters “B” and “A” are simply exchanged

**Fig. 3:**
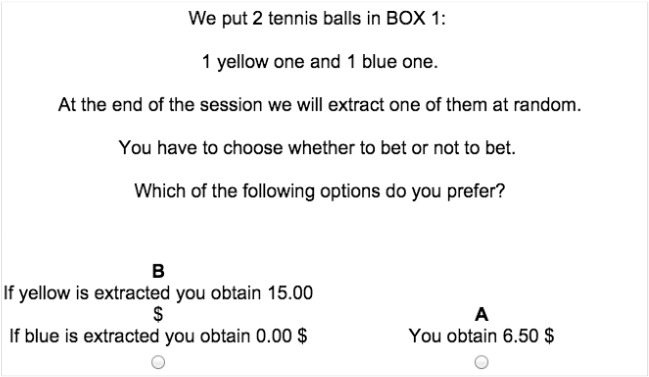
Screenshot of the lottery condition. As for the coordination games, in the lottery condition, participants were to choose between an uncertain option (“B”) and a lower paying but certain alternative (“A”). The outcome of the uncertain option depended on the extraction of a lottery containing a 1 “winning ball” and 1 “losing one”

A mixed effect logistic regression (Model 1, Table 3) reveals that friendship significantly interacts with the game environment by increasing the log odds of a UP action in the stag-hunt (Stag-hunt Game*Friend Counterpart equal to 1.732) while decreasing it in the entry game (*Friend Counterpart* equal to − 0.658). These effects appear very stark in Fig. 1, which builds on this logistic regression.

As illustrated in Fig. 1, friends are more likely to choose the UP action than strangers in stag-hunt games; by contrast, friends seem less likely to do so in entry games (Model 1, Table 3); these effects are also reliably observed when controlling for individual differences in risk attitudes (Model 2, Table 3). The empirical patterns shown in Fig. 1 also demonstrate that participants react as expected to the game incentives (e.g., deviation costs) in that increasing sure payoffs decrease the probability of UP actions. This impact of sure payoffs further interacts with friendship, such that the difference between friends and strangers increases with sure payoffs in the stag-hunt games, but decreases with sure payoffs in the entry games. Correspondingly, the effects of friendship are prominent at opposite sure payoff ranges in the two games, namely at low sure payoffs in the entry game and at high sure payoffs in the stag-hunt. This is unsurprising given that these sure payoff ranges are where normally coordination failures are more likely amongst strangers (Chierchia et al. 2018; Heinemann et al. 2009; Nagel et al. 2017), thus, introducing greater leeway to detect any friendship effect.

In Table 4, further econometric analysis suggests that the effect of friendship on choices is partially mediated by friendship quality, which significantly interacts with the game environment in determining the log odds of UP choices. Specifically, friendship quality has no impact on the odds of UP choices in entry games, but increases these odds in stag-hunts. In line with this, simple correlational analyses suggests that friendship quality is associated with a greater proportion of UP actions when participants interact with their friends in stag-hunt games (Spearman *ρ* = 0.371, *p* < 0.01), while it has no association with the corresponding measure in entry games (Spearman *ρ* = − 0.061, *p* = 0.596). The same finding is also robust when controlling for other friendship characteristics such as friends’ perceived interpersonal similarities and the frequency of their past interactions (Model 2 and 3, Table 4). Specifically, perceived similarities decrease the log odds of UP choices in entry games, while the frequency of past interactions interacts with the game environment in predicting choices. Correlational analysis suggests that perceived similarity among friends is negatively associated with UP actions when friends coordinate in entry games (Spearman *ρ* = − 0.272, *p* = 0.016), but not in stag-hunt games (Spearman *ρ* = − 0.132, *p* = 0.250), while the correlations between the frequency of past interactions and UP choices are not significant in either game (stag-hunt game: Spearman *ρ* = 0.133, *p* = 0.246; entry game: Spearman *ρ* = − 0.079, *p* = 0.494). In synthesis, friendship quality appears to play a role in fostering “assurance” in stag-hunt games; perceived interpersonal similarities could deter friends from entering in entry games, while frequency of past interactions has a small negative impact in stag-hunt games that does not appear robust in light of correlational analysis.

### Friendship and prosocial behaviour

**Fig. 4:**
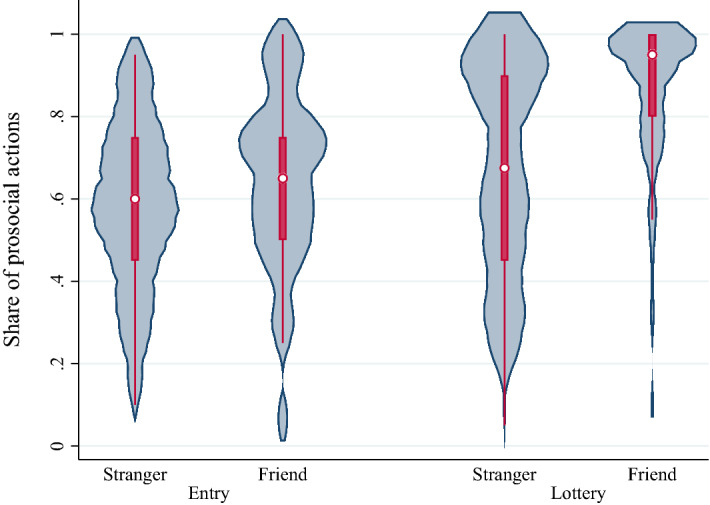
Impact of friendship on prosocial actions. There are four violin plots. Each plot includes a marker for the median, a box representing the interquartile range, spikes for the upper and lower adjacent values, and an overlaid estimated kernel density of the data at different values.

A mixed logistic effect model on prosocial behaviour (i.e., we code the variable *prosocial actions* as 1 if UP choice in the stag-hunt or SP choice in the entry game and 0 otherwise) shows a highly significant interaction between friendship and the game environment (Table 5, Appendix C): friendship significantly increases the log odds of prosocial actions in both the stag-hunt and the entry game; however, the effect is significantly reduced in the entry game, as suggested by the significant interaction between friendship and game. Consistently, Fig. 4 (Appendix C) shows stronger effects of friendship on prosocial actions in stag-hunt game than in entry games.

### Friendship and trembling

Mixed logistic effect models on trembling (Table 6, Appendix C) reveal a significant two-way interaction between the game environment and friendship, suggesting that friendship differentially affects the likelihood that participants would switch back and forth between actions in the two games: in the stag-hunts, participants are more likely to “tremble” when playing with strangers rather than friends. Conversely, relative to strangers, in the entry games, friendship raises the likelihood of switching one’s choice, albeit non-significantly.

### Friendship, coordination, and earnings

OLS regression models (Table 7, Appendix C) further show that friendship interacts with the game environment in affecting coordination and payoff rates. Specifically, friendship benefits both measures in the stag-hunt game, but these advantages are strongly decreased when passing to entry games.

## Discussion

In this study, we investigate how friendship affects coordination across experimental games of strategic complements and substitutes. We find that, relative to strangers, friends are more likely to choose UP actions in games of strategic complements (i.e., stag-hunt games). Conversely, relative to strangers, friends are more likely to choose the SP actions in games with strategic substitutes (i.e., entry games). In other words, friendship is associated with decreased strategic uncertainty in stag-hunts, but increased strategic uncertainty in entry games. These friendship effects are more pronounced at high sure payoff in stag-hunt games, and at low sure payoff in the entry game. In addition, we find that, in stag-hunts, friends tremble less than strangers (i.e., they display less violations of monotonicity); they are also more likely to successfully coordinate their choices and to earn more than strangers. However, each of these advantages is strongly attenuated or lost entirely in entry games.

Previous research suggests that social closeness generally has a beneficial impact on economic interactions (Sect. 1). Our experimental evidence adds to that literature by showing that such an impact depends on whether the interactions involve strategic complements or substitutes. In particular, this evidence challenges the intuitive notion that social closeness is always beneficial in situations involving tacit coordination.

We speculate that two broad mechanisms may mediate these effects: prosocial preferences and social inferences. In fact, friends are known to care about each other’s payoffs more than strangers, namely prosocial preferences are generally higher among friends than strangers (Jones and Rachlin 2006). Moreover, the payoff transformations of the games investigated here suggest that, in the stag-hunt game, friends could choose the UP action more frequently than strangers, because they want their friends not to lose out; conversely, in the entry game, friends could choose the UP action less frequently than strangers, because they want to avoid actions that could damage their friends. Accordingly, when recoding actions as choices between a prosocial and a non-prosocial action (rather than choices between UP and SP actions), we find that friendship increases the probability of prosocial actions in both games. However, we also find that the impact of friendship on prosocial actions is asymmetric across the games, in that it is reduced in entry games. We thus suspect that while prosocial motives (e.g., Camerer 2003; Fehr and Fischbacher 2003; Lange 1999) may contribute to our findings, they are unlikely to explain them entirely.

On the other hand, the notion of social distance as the “degree of reciprocity that subjects *believe* exist in a social interaction” (Hoffman et al. 1996—italics added) appears intrinsically inferential as it taps on beliefs. Various well-documented effects of social closeness on economic interactions, along dimensions such as group membership, have been suggested to primarily rely on beliefs (Guala et al. 2013). Even among strangers, such “reciprocal expectations” have been long documented in games of economic exchange (Fischer 2009; Rubinstein and Salant 2016) and evolutionary simulations with reciprocal expectations appear to outperform a number of alternative models in repeated social dilemmas (Fischer et al. 2013). Moreover, this is psychologically plausible given that is well known that humans frequently resort to their own thoughts and preferences—and even recruit the same neural structures—to make inferences about others, *especially* when they are perceived as “close”, in-groups or similar to themselves (Ames 2004; Robbins and Krueger 2005; Denny et al. 2012; Benoit et al. 2010). Indeed, if agents expect that their friends are more likely than strangers to reciprocate their actions, this could provide assurance in stag-hunts with an increase in the likelihood of UP actions, but deterrence from choosing the same action in entry games with the consequent reduction in the likelihood of UP actions.

To some extent, the “shadow of the future” in the form of possibility to retaliate and willingness to share money are, for instance, plausibly inherent constituents of friendship. Given the early stage of research on friendship and economic interactions, we here opted to investigate full-fledged friendship. Moreover, to control for the possible shadow of the future effects of friendship, we used the frequency of past interactions as a proxy for future interactions (Zhang 2001). We find that the latter has a weak differential effect in the two games, but that it is far from over-shadowing the effects of friendship quality and similarity on coordination. Future studies could attempt to isolate the separate contributions of friendship constituents on coordination. For example, it should be possible to engineer a stranger treatment with a built-in possibility for participants to share experimental money; or with opportunities for “retaliation” in subsequent experimental games/interactions.

## Conclusions

We find that, relative to strangers, friendship is associated with decreased strategic uncertainty in games with strategic complements (i.e., two-player stag-hunt game), but with increased strategic uncertainty in games with strategic substitutes (i.e., two-player entry game). With regard to other performance proxies, such as trembling, coordination, and earnings, friendship has a substantially beneficial impact on games with strategic complements, but these advantages are either attenuated or entirely lost in games with substitutes. Our evidence adds to the fast-growing literature on the importance of the social context in explaining economic behaviour (Charness et al. 2007a, b; Chen and Chen 2011; Kranton 2016; Chierchia and Coricelli 2015; Gächter et al. 2019) and illustrates the scope for exploring the differential role of friendship across a variety of strategic situations ranging from pure coordination games, through mixed-motive games, to pure conflictual (i.e., zero-sum) games.

From an applied viewpoint, our findings have also potentially relevant implications for understanding team production in organizations. In fact, within organizations, as well as in many daily interactions, decision-makers *are* embedded in a social space, and they constantly tune their behaviour onto the “social closeness” of others. Moreover, their interactions may involve either strategic complements or substitutes depending on members’ roles, tasks, skills, and objectives. Our findings provide preliminary insight that friendship need not equally benefit all types of tasks. Finally, from a theoretical viewpoint, these findings may prompt new developments for modelling behaviour in strategic interactions across families of games and social contexts, thus tackling the question of how well-known gradients in the social space (here dichotomized as friends vs. strangers) may interact with well-known gradients in the “strategic space” (here dichotomized as complementarity vs. substitutability) on a larger scale.

## Figures and Tables

**Fig. 5:**
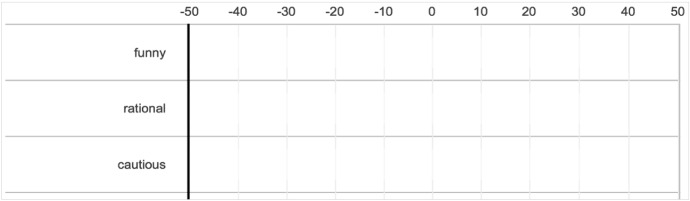
Screenshot of a section of the similarity questionnaire. The complete list of words is: smart, opinionated, driven, cooperative, competitive, self-critical, fun, funny, opinionated, lively, self­controlled, authoritative, disciplined, altruistic, idealistic, rational, generous, dynamic, cautious, and honest. Participants are instructed to rate each of these words twice. Once they are instructed the following: “If "0" represents the AVERAGE USC student, how much does each of the following traits represent ME? (A negative sign means a given trait represents you “less” than the average USC student, a “positive” sign that a given trait represents you more than the average USC student).” The second time, they are instructed the following: “If “0” represents the AVERAGE USC student, how much does each of the following traits represent [NAME OF PARTICIPANT’S FRIEND]? (Your answers will NOT be revealed to your friend, or anyone else, simply answer as honestly as possible).” The order of words is randomly shuffled in each of these two blocks. The Pearson’s correlation of self and friend ratings is used a perceived similarity measure

## Appendix B

See Figs. 2, 3.

## Appendix C

See Table 5

**Table 5:** Prosocial choices: mixed-effects logistic regressions

Estimation method: mixed-effects logistic Controls for individual effects: clustering Dependent variable: prosocial choice (dummy)	
Sure payoff	0.231*** (0.014)
Stag-hunt game (dummy)	4.849*** (0.184)
Stag-hunt game*Sure payoff	− 0.556*** (0.019)
Friend counterpart (dummy)	0.478*** (0.151)
Friend counterpart*Sure payoff	− 0.024 (0.018)
Stag-hunt game*Friend counterpart	1.235*** (0.147)
Constant	− 1.344*** (0.146)
No. observations	6196
No. individuals	78
Log-likelihood	− 2989.958

(Robust) Standard errors are in parentheses

***Significance at the 1% level

See Fig. 4

See Tables 6, 7

**Table 6:** Trembling and friendship: mixed-effects logistic regressions

Estimation method: mixed-effects logistic Controls for individual effects: clustering Dependent variable: threshold (dummy)	
Sure payoff	− 0.066*** (0.015)
Stag-hunt game (dummy)	− 2.415*** (0.198)
Stag-hunt game*Sure payoff	0.178*** (0.020)
Friend counterpart (dummy)	− 0.066 (0.164)
Friend counterpart*Sure payoff	0.011 (0.018)
Stag-hunt game*Friend counterpart	− 0.366** (0.152)
Constant	− 0.578*** (0.142)
No. observations	5848
No. individuals	78
Log-likelihood	− 2615.490

(Robust) Standard errors are in parentheses

***Significance at the 1 percent level

**Significance at the 5 percent level

**Table 7:** Coordination and earnings: OLS regressions

Estimation method: ordinary least square		
Controls for individual effects: clustering		
	Model 1	Model 2
Dependent variable:	Coordination rates	Payoff rates
Stag-hunt game	0.251*** (0.011)	2.675*** (0.098)
Friend counterpart	0.047** (0.022)	0.613*** (0.204)
Stag-hunt game*Friend counterpart	0.097*** (0.027)	1.400*** (0.319)
Constant	0.411*** (0.008)	8.302*** (0.040)
No. observations	312	312
No. individuals	78	78
*R*^2^	0.568	0.604

(Robust) Standard errors are in parentheses

***Significance at the 1 percent level

**Significance at the 5 percent level

## Appendix D

See Fig. 5

## Footnotes

1 Notable exceptions are Charness et al. (2007a, b), Cobo-Reyes and Jiménez (2012), Chen and Chen (2011), and Gächter et al. (2019). Charness et al. (2007a, b) prove how salient group membership increases coordination rates in a battle of the sexes game. Cobo-Reyes and Jiménez show the efficiency-reducing effect of social closeness in two novel asymmetric coordination games. Chen and Chen (2011) evidence how lab-induced group identity improves coordination in a two-player weak-link game with a continous action space. Gächter et al. (2019) demonstrate the efficiency-enhancing effect of pre-existing social closeness in a four-player weak-link coordination game with five possible actions.

2 For instance, this is the case for proximity in social network (Apicella et al. 2012), artificial group membership (Balliet, Wu, & Dreu, 2014 for a meta-analysis), natural group membership (Bernhard, Fischbacher, and Fehr 2006), social identification (Hoffman, McCabe, and Smith 1996), motor synchronization (Wiltermuth and Heath 2009), and interpersonal similarity (Cole and Teboul 2004).

3 The experiment was computerised in Qualtrics (Qualtrics, Povo). Full instructions are available in Appendix A.

4 In addition, participants took part in a second identical lottery task in which *p* was unknown to participants. This was done only for replication purposes (Chierchia, Nagel, and Coricelli 2018) and is not discussed further.

5 Since the MFQ subscales were all highly inter-correlated (all correlations: Spearman ρ > 0.729, p < 0.001), we aggregate across all items and take this resulting average MFQ score as a general measure of friendship quality (henceforth *MFQ index*).

6 The perceived similarity measures are on average positive (mean = 0.284; std. dev. = 0.338) and imply that friends believe they are more likely than the average student from their own University (the “stranger” in our experiment) to be described by the same traits (one-sample t-test against a zero mean: t(77) = 7.425; p < 0.001). Moreover, our similarity measure does not predict general friendship quality (Spearman ρ = 0.045, p = 0.696), as measured by the MFQ index.

7 Unsurprisingly, the MFQ index correlates with the frequency of past interactions among friends (Spearman ρ = 0.519, p < 0.001).

8 Fifty-five percent of our participants were females, with an overall sample average age of 20.5 years (std. dev.: 3.1).
